# Bitter taste receptor agonists induce vasorelaxation in porcine coronary arteries

**DOI:** 10.3389/fphar.2025.1578913

**Published:** 2025-07-07

**Authors:** Ching-Chung Tsai, Yi-Chen Li, Li-Ching Chang, Shih-Che Huang

**Affiliations:** ^1^ Department of Pediatrics, E-Da Hospital, I-Shou University, Kaohsiung, Taiwan; ^2^ School of Medicine, I-Shou University, Kaohsiung, Taiwan; ^3^ Department of Medical Research, E-Da Hospital, Kaohsiung, Taiwan; ^4^ Department of Internal Medicine, Ikutora Clinic, Hokkaido, Japan

**Keywords:** TAS2R agonists, vasorelaxation, ischemic heart disease, bitter taste receptors, porcine coronary arteries, nitric oxide signaling, potassium channels

## Abstract

**Background:**

Ischemic heart disease (IHD) remains a leading cause of global morbidity and mortality, necessitating the search for novel therapeutic approaches. Recent studies have identified bitter taste receptors (TAS2Rs) in vascular smooth muscle cells as potential therapeutic targets because of their vasorelaxant properties. This study investigated the vasorelaxant effects of TAS2R agonists on porcine coronary arteries *ex vivo* and explored their potential as novel therapeutic targets for IHD.

**Methods:**

Isolated porcine coronary artery rings were precontracted using U46619 and treated with TAS2R agonists, including flufenamic acid, dapsone, phenanthroline, chloroquine, and quinine. Vasorelaxation induced by TAS2R agonists was quantitatively assessed, and pharmacological inhibitors were used to elucidate the underlying mechanisms of vasorelaxation. Real-time PCR analysis was conducted to confirm the expression of specific TAS2R subtypes in porcine coronary arterial tissue.

**Results:**

TAS2R agonists induced concentration-dependent vasorelaxation, with flufenamic acid showing potent effects, exhibiting an EC50 of 30.4 μM, whereas phenanthroline and chloroquine exhibited moderate responses. In contrast, quinine and dapsone showed mild relaxation. The flufenamic acid-induced effect was attenuated by NG-nitro-L-arginine (47.4% ± 3.04%), apamin (49.2% ± 3.7%), and glibenclamide (49.6% ± 1.5%), indicating the involvement of nitric oxide signaling and potassium channels. PCR analysis revealed the differential expression of TAS2R subtypes, with TAS2R42 showing the highest expression, followed by subtypes 40, 10, and 38.

**Conclusion:**

This study showed that TAS2R agonists, especially flufenamic acid, phenanthroline, and chloroquine, induced vasorelaxation in isolated porcine coronary arteries. The vasorelaxation mechanism of flufenamic acid may involve nitric oxide signaling and potassium channels. The expression of specific TAS2R subtypes, together with functional observations, suggest that bitter taste receptors play a role in coronary vascular regulation, warranting further investigation into their therapeutic potential.

## 1 Introduction

Ischemic heart disease (IHD), also known as coronary artery disease (CAD), is a leading cause of morbidity and mortality globally (Khan et al., 2020). It arises from reduced blood flow to the heart, primarily from coronary artery narrowing or blockage by an atherosclerotic plaque (Noothi et al., 2023). The clinical presentations of IHD range from stable angina, characterized by exertional chest pain owing to myocardial oxygen demand-supply mismatch, to acute coronary syndromes, including myocardial infarction caused by plaque rupture and thrombosis (Palasubramaniam et al., 2019). Timely intervention is critical for preventing severe complications such as heart failure and sudden cardiac death (Jahmunah et al., 2021). Management of IHD involves a multifaceted approach combining lifestyle modifications, pharmacological interventions, and, in appropriate cases, revascularization procedures (Boden et al., 2023). Although conventional pharmacological treatments, including antiplatelet agents, beta-blockers, statins, and angiotensin-converting enzyme inhibitors have demonstrated cardiovascular benefits, challenges regarding symptom control and adverse effects (Virani et al., 2023; American Diabetes Association Professional Practice Committee, 2024). These limitations highlight the need for novel therapeutic approaches (Arnold et al., 2020; Bansal and Hiwale, 2023; Nanna et al., 2018).

Bitter taste receptors (type 2 taste receptors, TAS2Rs) have emerged as potential therapeutic targets, as their functions extend well beyond taste perception to wider physiological processes, including cardiovascular regulation. These G protein-coupled receptors (GPCRs) function within the complex system of human taste perception and encompass five primary tastes: sweet, salty, umami, bitter, and sour. Within this system, TAS1Rs mediate sweet and umami taste perception, whereas TAS2Rs, with over 25 identified variants in humans, primarily serve as sensors for bitter compounds (Bachmanov et al., 2014; Ahmad and Dalziel, 2020; Jaggupilli et al., 2016).

TAS2Rs are widely expressed in non-gustatory tissues, including the nervous system, lungs, and immune cells, where they regulate inflammatory cytokine production and airway smooth muscle tone (Grassin-Delyle et al., 2014; D'Urso and Drago, 2021). In the gastrointestinal tract, these receptors influence appetite and gut hormone secretion (Descamps-Solà et al., 2023; Avau and Depoortere, 2016), whereas in reproductive tissues, they mediate processes such as sperm chemotaxis, and potentially prevent preterm birth (Lu et al., 2024).

Although research has begun to uncover the involvement of TAS2Rs in cardiovascular function (Welcome et al., 2023; Bloxham et al., 2020; Manson et al., 2014; Chen et al., 2017; Bloxham et al., 2024), their specific roles in coronary artery regulation and therapeutic potential in IHD remain underexplored. Given the anatomical and physiological similarities between porcine and human vascular systems, porcine coronary arteries were selected for this study. Recent evidence suggests that pigs are superior to rodents and rabbits in replicating human arterial pathophysiology (Cupitra et al., 2023). This study evaluated the vasorelaxant effects of TAS2R agonists on porcine coronary arteries and elucidated the underlying mechanisms, potentially paving the way for innovative cardiovascular therapies.

## 2 Materials and methods

### 2.1 Material acquisition and preservation

Given the anatomical similarities between human and porcine coronary arteries, the latter are effectively used as models to study human coronary vascular function (Suzuki et al., 2011). All procedures were performed in accordance with the relevant laws and institutional guidelines of the E-Da Hospital. Porcine hearts were obtained from healthy pigs, each weighing approximately 110 kg, raised for consumption and not specifically for experimental purposes. Pigs were humanely euthanized at Bao-Yi Frozen Food Co., Ltd., a licensed local slaughterhouse located in Pingtung County, Taiwan, using electrical stunning and exsanguination under government-approved procedures. Typically, 2–3 porcine hearts were obtained per acquisition session, depending on availability. The hearts were immediately immersed in ice-cold Krebs-Henseleit buffer and transported to the laboratory within approximately 30 min in insulated containers to ensure tissue viability. All experiments were conducted using fresh tissues within approximately 50 min of harvest (The heart collecting and processing time, from slaughtering, was within approximately 20 min and transportation time was within approximately 30 min) to ensure tissue viability. The Krebs-Henseleit buffer solution consisted of 122 mM NaCl, 4.7 mM KCl, 15.5 mM NaHCO_3_, 1.2 mM KH_2_PO_4_, 1.2 mM MgCl_2_, 1.8 mM CaCl_2_, and 11.5 mM glucose, with the pH adjusted to 7.4. The solution was oxygenated with 95% O_2_ and 5% CO_2_ for at least 15 min before specimen collection. Although this study was exempt from requiring formal approval by the Institutional Animal Care and Use Committee (IACUC) because of the use of agricultural animals not specifically raised for experimental purposes, the study protocol was reviewed by the IACUC of E-Da Hospital, and an exemption certificate was officially issued on 24 January 2025. All procedures involving animal tissues were conducted in strict accordance with the ethical standards for animal tissue research. The pharmacological agents used in this study, including phenanthroline, flufenamic acid, dapsone, chloroquine, quinine, denatonium benzoate, U46619, apamin, KT5720, KT5823, and NG-nitro-L-arginine (L-NNA), were purchased from Sigma-Aldrich (St. Louis, MO, United States). Additional agents included rolipram, vardenafil, and tetraethylammonium (TEA) from Santa Cruz Biotechnology (Santa Cruz, CA, United States), iberiotoxin (IbTX) from Alomone Labs (Jerusalem, Israel), glibenclamide from Research Biochemicals International (Natick, MA, United States), and tetrodotoxin (TTX) from Tocris Bioscience (Bristol, United Kingdom).

### 2.2 Isolation and preparation of porcine coronary artery rings

Porcine coronary arteries were prepared according to previously published procedures, with minor modifications (Wang et al., 2016; White et al., 2005). Upon arrival at the laboratory, the epicardium, excess fat, and connective tissues were carefully removed from each heart using a combination of slow, deliberate snipping with scissors and careful separation with forceps. The left anterior descending coronary artery was subsequently dissected. The arteries were sectioned into rings approximately 5 mm in width. Four rings were obtained for each coronary artery. The endothelium was carefully removed by gently rubbing the luminal surface to mitigate the potential indirect effects of endothelium-derived vasoactive factors. The prepared arterial rings were mounted between a small metal rod attached to a fixed support and a triangular hook (Radnoti, Monrovia, CA, United States) in organ baths filled with Krebs-Henseleit buffer. The buffer was maintained at 37°C and continuously oxygenated with 95% O_2_ and 5% CO_2_. The pH was 7.40 ± 0.05. The triangular hook was connected to an isometric force transducer (FORT10g; World Precision Instruments, Sarasota, FL, United States) via a surgical silk thread for tension measurements. The transducer signals were amplified using a biological signal amplifier (MP36, BIOPAC Systems, Santa Barbara, CA, United States) and recorded using computer system software (BSL PRO 3.7.3, BIOPAC Systems, Santa Barbara, CA, United States). An optimal resting tension of 2.0 g was applied and the buffer was refreshed every 30 min. After initial equilibration, the rings were exposed to 60 mM KCl Krebs-Henseleit buffer for 4 min to induce baseline contractile activity and test the physiological viability of the vascular rings.

### 2.3 Exposure to bitter taste receptor agonists

After removing the 60 mM KCl Krebs-Henseleit buffer, the tissue was washed three times with fresh Krebs-Henseleit buffer. Subsequently, a 45-min equilibration period was observed to allow the tissues to stabilize. Subsequently, the coronary artery rings were contracted using 100 nM U46619, a thromboxane A2 analog, to induce stable precontraction (Hu et al., 2012). Upon achieving stable contraction, the rings were exposed to various concentrations of bitter taste receptor agonists. Flufenamic acid, phenanthroline, and dapsone were tested separately at individual concentrations of 10, 30, 100, and 300 μM. The half-maximal effective concentration (EC_50_) was defined as the concentration of a compound producing 50% of its maximal response. Based on the EC_50_ value of flufenamic acid (30.4 μM) determined from concentration–response experiments, 30 μM was selected for subsequent mechanistic studies. Similarly, chloroquine, quinine, and denatonium benzoate were each tested at individual concentrations of 30, 100, and 300 μM. This was performed using a non-cumulative method to ascertain their effects on coronary artery relaxation. Relaxation responses were represented as a percentage of U46619-stimulated contractions. Only a single-dose response was observed for each preparation (Hu et al., 2012).

### 2.4 Influence of neuronal conduction on relaxation induced by flufenamic acid

We investigated the potential role of neuronal conduction in flufenamic acid-mediated vasorelaxation. The experimental protocol involved pre-treating with 1 μM TTX, a selective blocker of neuronal sodium channels, for 15 min before the addition of 30 μM flufenamic acid. This was performed after pre-inducing contractions with 100 nM U46619 (Tsai et al., 2018b).

### 2.5 Impact of rolipram and vardenafil on relaxation promoted by flufenamic acid

We examined the potentiation of flufenamic acid-induced vasorelaxation via cyclic nucleotide signaling pathways. The experimental protocol involved using two inhibitors: 1 μM rolipram, a selective phosphodiesterase-4 (PDE-4) inhibitor to elevate cAMP levels, and 1 μM vardenafil, a phosphodiesterase-5 (PDE-5) inhibitor to increase cGMP levels. Coronary artery rings were pre-treated with either inhibitor for 20 min, then exposed to 30 μM flufenamic acid following precontraction induced by 100 nM U46619 (Tsai et al., 2018a).

### 2.6 Implications of cAMP, cGMP, and nitric oxide (NO) in flufenamic acid-induced relaxation

We investigated the contributions of cAMP, cGMP, and nitric oxide to the vasorelaxant effects of flufenamic acid. Specific inhibitors were employed to elucidate the roles of these mediators: 1 μM KT5720 to inhibit cAMP-dependent protein kinase (PKA), 1 μM KT5823 to inhibit cGMP-dependent protein kinase (PKG), and 100 μM L-NNA to inhibit nitric oxide synthase. Coronary artery rings were incubated with each inhibitor for 30 min before the addition of 30 μM flufenamic acid, following precontraction induced by 100 nM U46619 (Tsai et al., 2018a; Tsai et al., 2018b).

### 2.7 Exploration of potassium channels in flufenamic acid-mediated vasorelaxation

Given their critical function in regulating vascular smooth muscle tone, we aimed to elucidate the role of potassium channels in flufenamic acid-induced vasorelaxation. Various potassium channel inhibitors were employed: 1 mM TEA, primarily targeting large-conductance calcium-activated potassium channels (Barman et al., 2004); 100 nM apamin, targeting small-conductance calcium-activated potassium (SK_Ca) channels; 200 nM IbTX, focused on large conductance calcium-activated potassium channels; and 10 μM glibenclamide, inhibiting ATP-sensitive potassium channels. Each inhibitor was introduced 30 min before the administration of 30 μM flufenamic acid, following precontraction induced by 100 nM U46619, to determine the specific channels contributing to the vasorelaxation (Tsai et al., 2018a; Tsai et al., 2018b).

### 2.8 Analysis of TAS2Rs mRNA expression in porcine coronary artery tissues

To investigate TAS2Rs mRNA expression in porcine coronary artery tissues, total RNA was extracted using RNA Isolater Total RNA Extraction Reagent (Vazyme Biotech, Nanjing, China) according to the manufacturer’s instructions. RNA concentration and purity were assessed using an Epoch spectrophotometer (BioTek Instruments, Winooski, United States).

cDNA was synthesized from 1,000 ng of total RNA using HiScript III RT SuperMix (Vazyme Biotech, Nanjing, China) according to the supplier’s protocol. Quantitative real-time PCR (qPCR) was performed using the StepOne Plus system (Applied Biosystems, Foster City, CA, United States) to quantify the mRNA levels of all sequences of the porcine TAS2R subtypes, including TAS2R 3, 4, 7, 8, 9, 10, 16, 38, 40, 41, 42, and 60. TAS2R14 was not included in our qPCR panel because of the absence of available sequence information in genetic databases. GAPDH and β-actin were used as reference genes. Relative expression was calculated using the 2^−ΔΔCT^ method, normalized to the geometric mean of GAPDH and ACTB (Schmittgen and Livak, 2008; Tsai et al., 2019). The primer design for porcine TAS2Rs was based on sequences obtained from the NCBI database. The primer sequences and validation details are provided in Supplementary Table S1.

### 2.9 Statistical analysis

Data are presented as mean ± SEM. Statistical comparisons were performed using Student’s t-test for two-group comparisons or one-way analysis of variance (ANOVA) followed by Dunnett’s multiple comparison test for comparisons between more than two groups. *p*-values <0.05 were considered statistically significant. All statistical analyses were conducted using SPSS software, version 24 (IBM Corp., Armonk, NY, United States), and GraphPad Prism 10.0 (GraphPad Software, San Diego, CA, United States) was used to determine the EC50 values.

## 3 Results

### 3.1 Evaluation of bitter receptor agonists on coronary arterial relaxation following U46619-Induced contractions

**FIGURE 1:**
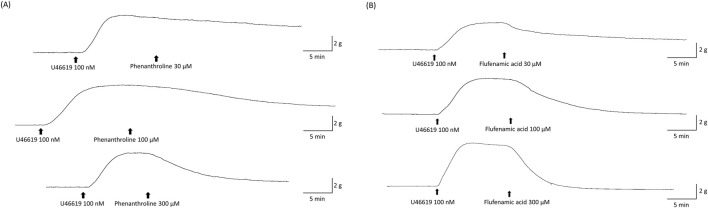
Representative relaxant tracings of phenanthroline **(A)** and flufenamic acid **(B)** in U46619-precontracted porcine coronary artery segments. Initially, each artery segment was precontracted using 100 nM U46619. The tracings detail the vasorelaxation induced by phenanthroline at doses of 30, 100, and 300 μM **(A)** and flufenamic acid at concentrations of 30, 100, and 300 μM **(B)**, respectively. The arrows indicate the addition of U46619, phenanthroline **(A)**, and flufenamic acid **(B)**.

We evaluated the effects of bitter taste receptor agonists, including phenanthroline, flufenamic acid, dapsone, denatonium benzoate, chloroquine, and quinine, on the vasorelaxation of U46619-precontracted coronary arterial segments. Concentration-dependent responses were recorded for phenanthroline and flufenamic acid, illustrated in Figures 1A,B, across concentrations from 30 to 300 μM.

**FIGURE 2:**
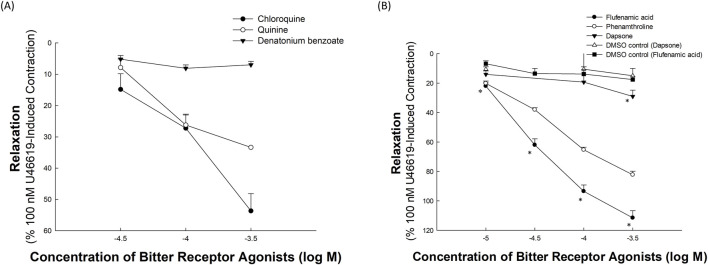
Relaxation effects of bitter receptor agonists on porcine coronary artery segments. These effects were measured after exposure to different bitter receptor agonists, following an initial contraction induced by 100 nM U46619. **(A)** Relaxation responses to chloroquine, quinine, and denatonium benzoate, clearly illustrate the varying efficacies of these compounds in inducing relaxation in the artery segments. **(B)** Effects of flufenamic acid, phenanthroline, and dapsone. This panel also compares the relaxation effects of these substances with their respective dimethyl sulfoxide (DMSO) controls to evaluate the specific impact of the drugs versus the solvent used. Data points represent the mean relaxation response, with error bars showing the standard error of the mean (SEM). The values are expressed as a percent of a U46619 (100 nM)-induced contraction. The results given are from at least five independent experiments. An asterisk (*) denotes a statistically significant difference from the vehicle (DMSO) control (*p* < 0.05).

Figure 2A shows the relaxation responses of chloroquine, quinine, and denatonium benzoate to U46619-induced precontractions. Chloroquine and quinine exhibited concentration-dependent relaxation effects, with chloroquine producing a more pronounced response at higher concentrations. In contrast, denatonium benzoate showed a minimal relaxation effect across all tested concentrations. Each data point represents at least five independent experiments (n ≥ 5 per group).

Figure 2B illustrates the relaxation effects of flufenamic acid, phenanthroline, and dapsone following the U46619-induced precontraction of porcine coronary artery rings. Flufenamic acid exhibited the most potent, concentration-dependent relaxation, increasing from 22.01% ± 2.19% at 10 μM to 111.53% ± 4.82% at 300 μM. Phenanthroline also produced a potent, dose-dependent response, ranging from 20.13% ± 1.57% at 10 μM to 82.28% ± 2.47% at 300 μM. In contrast, dapsone showed mild relaxation (14.02% ± 2.31% at 10 μM to 29.03% ± 4.25% at 300 μM). The relaxation induced by flufenamic acid was statistically significant at all tested concentrations compared with the DMSO control (*p* < 0.05), whereas dapsone showed a significant difference only at the highest concentration (300 μM). Each data point represents at least five independent experiments (n ≥ 5 per group).

The estimated EC_50_ for flufenamic acid causing relaxation was 30.4 μM. Given its pronounced relaxation effects, flufenamic acid at 30 μM was selected for further investigation on its vasorelaxation mechanisms in the coronary arteries.

### 3.2 Neuronal Conduction’s influence on flufenamic acid-induced relaxation in porcine coronary arteries

**FIGURE 3:**
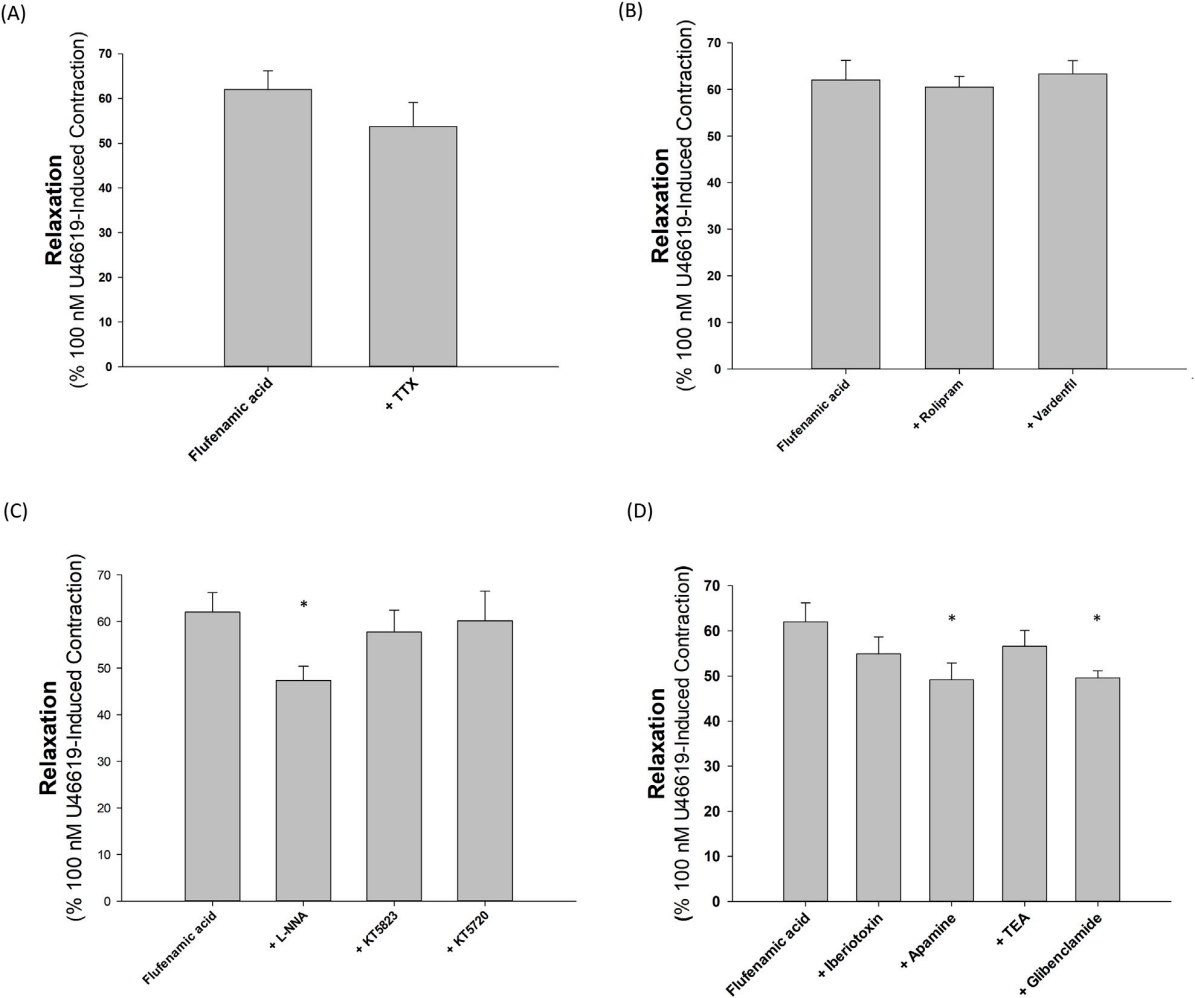
Inhibition of various agents on vasorelaxation induced by flufenamic acid in segments of the porcine coronary artery precontracted with 100 nM U46619. **(A)** The presence of tetrodotoxin (TTX) did not significantly alter the vasorelaxant action of flufenamic acid. **(B)** The addition of rolipram and vardenafil also did not significantly modify the vasorelaxation induced by flufenamic acid. **(C)** Neither KT5720 nor KT5823 significantly affected the vasorelaxant response to flufenamic acid, whereas NG-nitro-L-arginine (L-NNA) significantly inhibited the flufenamic acid-induced vasorelaxation. **(D)** Iberiotoxin (IbTX) and tetraethylammonium (TEA) had no significant impact on the relaxation response to flufenamic acid, but apamin and glibenclamide significantly decreased the relaxation effect. Error bars represent the standard error of the mean (SEM). The single asterisk (*) denotes a statistically significant deviation from the relaxation effect at 30 μM flufenamic acid, with *p* < 0.05.

As illustrated by Figure 3A, the relaxant capacity of 30 μM flufenamic acid on U46619-precontracted porcine coronary arteries remained unaffected by the application of 1 μM TTX (*p* > 0.05, n = 5).

### 3.3 Influence of phosphodiesterase inhibitors on flufenamic acid-induced relaxation in porcine coronary arteries

Figure 3B shows that incorporating 1 μM rolipram or 1 μM vardenafil did not enhance the relaxation effect of 30 μM flufenamic acid in U46619-precontracted porcine coronary arterial rings (*p* > 0.05, n = 5).

### 3.4 Role of nitric oxide and protein kinases in flufenamic acid-induced relaxation in porcine coronary arteries

As depicted in Figure 3C, the relaxation response to 30 μM flufenamic acid in U46619-precontracted porcine coronary arteries was reduced in the presence of 100 μM L-NNA (*p* < 0.05, n = 5). However, this response did not diminish in the presence of 1 μM KT5823 or 1 μM KT5720 (*p* > 0.05, n = 6 and 5, respectively).

### 3.5 Effects of potassium channels on flufenamic acid-induced relaxation of porcine coronary arteries

Figure 3D illustrates that 200 nM IbTX and 1 mM TEA did not suppress the relaxation induced by 30 μM flufenamic acid in U46619-precontracted porcine coronary arteries (*p* > 0.05, n = 5 and 6, respectively). Conversely, 100 nM apamin significantly decreased the relaxation effect (*p* < 0.05, n = 6), and 10 μM glibenclamide also reduced the relaxation prompted by 30 μM flufenamic acid (*p* < 0.05, n = 8).

### 3.6 Expression of bitter taste receptors in porcine coronary artery

**FIGURE 4:**
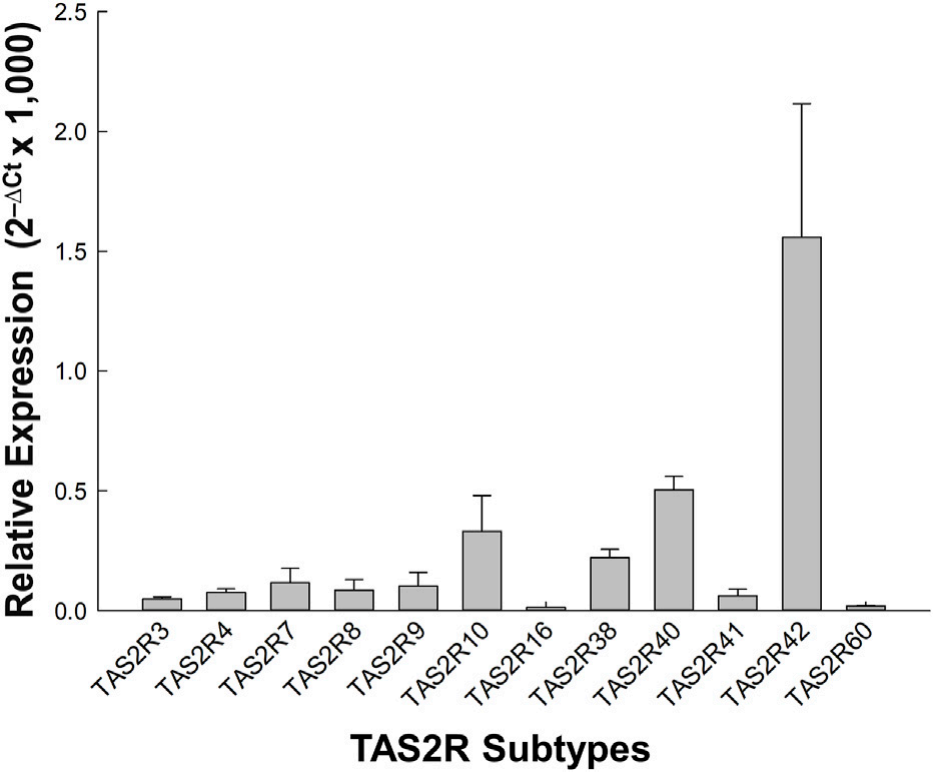
Relative expression of TAS2R subtypes in porcine coronary artery tissue. This figure shows the relative expression levels (2^−ΔCt^ × 1,000) of various TAS2R subtypes across three independent samples. The bar graph illustrates mean relative expression levels for each TAS2R subtype, with error bars representing the standard error of the mean. Data are normalized to the geometric mean of GAPDH and ACTB reference genes. TAS2R42 shows notably higher expression compared to other subtypes, suggesting a predominant role in the porcine coronary artery tissue. This is followed by TAS2R40, TAS2R10, and TAS2R38, indicating their potential importance in vascular responses mediated by bitter taste receptors.

As shown in Figure 4, real-time PCR analysis was performed to examine the expression of TAS2R subtypes in porcine coronary artery tissues. Among the subtypes, TAS2R42 exhibited the highest expression with a mean relative quantification (RQ) value of 1.559 ± 0.558, significantly higher than all other subtypes. This was followed by TAS2R40, TAS2R10, and TAS2R38, with RQ values of 0.504 ± 0.097, 0.332 ± 0.149, and 0.222 ± 0.035, respectively. Data are shown as means ± SEM from three independent samples (n = 3).

## 4 Discussion

TAS2Rs, traditionally recognized for the detection of bitter substances, play significant roles beyond taste perception, particularly in cardiovascular physiology. These receptors are expressed not only in taste-associated tissues but also in vascular smooth muscle cells and cardiac tissues, where they affect muscle contractility and vascular tone (Bloxham et al., 2020; Tuzim and Korolczuk, 2021). Our study contributes to this expanding knowledge by demonstrating that TAS2R agonists induced concentration-dependent vasorelaxation in porcine coronary arteries. In cardiac tissues, TAS2Rs act as molecular sentinels that detect harmful or pathogenic molecules and initiate protective responses that regulate crucial pathways involved in inflammation and oxidative stress, which are key processes in the prevention of heart diseases (Welcome et al., 2023; Bloxham et al., 2024). Additionally, recent findings have confirmed the expression of TAS2Rs in vascular smooth muscle cells across various systemic arteries, including the aorta and the mesenteric, cerebral, and pulmonary arteries, underscoring their extensive physiological roles in regulating vascular tone and blood flow (Manson et al., 2014; Chen et al., 2017; Liu et al., 2020). A recent study by Kochany et al. (2024), preprint article, have identified TAS2R subtypes in vascular endothelial cells, confirming mRNA expression of all 25 TAS2Rs and protein expression of TAS2R10 and TAS2R38 in human aortic and coronary artery endothelial cells via digital PCR and Western blot. Although preliminary, these findings further support the involvement of TAS2Rs in vascular physiology and highlight the need for further peer-reviewed validation. Collectively, these insights emphasize the therapeutic potential of TAS2R agonists for modulating vascular function and highlight innovative approaches for treating vascular disorders.

Our study revealed that TAS2R agonists, such as flufenamic acid, dapsone, phenanthroline, chloroquine, and quinine, significantly relaxed porcine coronary artery rings in a concentration-dependent manner. Flufenamic acid showed the most potent effect, whereas denatonium benzoate had a minimal impact. Identifying TAS2Rs as potential targets offers a unique approach beyond the current therapies for CAD. TAS2R agonists may complement existing treatments and offer novel options for patients with ischemic heart conditions.

Our investigation of the mechanisms underlying flufenamic acid-induced coronary vasorelaxation revealed that neuronal activity (tested using TTX), cyclic nucleotide pathways (examined using rolipram and vardenafil), and protein kinase pathways (assessed using KT5823 and KT5720) were not involved. However, nitric oxide (NO) signaling may play a crucial role, as evidenced by the diminished relaxation response when NO synthase was inhibited by L-NNA. Furthermore, we found that activation of specific potassium channels led to flufenamic acid-induced coronary vasorelaxation (Jackson, 2017). Although inhibitors, such as IbTX and TEA, had no significant impact on flufenamic acid-induced relaxation, apamin and glibenclamide significantly reduced relaxation. This indicates that flufenamic acid may affect small-conductance calcium-activated and ATP-sensitive potassium channels.

qPCR analysis revealed a complex interplay between TAS2R expression and functional outcomes in porcine coronary arteries. We found that the TAS2R subtypes 42, 40, 10, and 38 were specifically expressed in these arteries, with TAS2R42 showing the highest expression, followed by TAS2R40, TAS2R10, and TAS2R38. Immunohistochemical studies were not performed because of the lack of specific antibodies against porcine TAS2R subtypes 42, 40, 10, and 38.

These data revealed a complex interplay between TAS2R agonist-induced relaxation, receptor subtype specificity, and TAS2R expression in porcine coronary arteries. The effects of dapsone (targeting TAS2R40, TAS2R4, and TAS2R10) (Meyerhof et al., 2010; Grassin-Delyle et al., 2019) and chloroquine (targeting TAS2R3, TAS2R10, and TAS2R39) (Meyerhof et al., 2010; Navarro-Dorado et al., 2023; Zhang et al., 2020) were consistent with the high expression of TAS2R40 and TAS2R10 observed in our qPCR results, highlighting these subtypes as potential key mediators of coronary vasorelaxation. The substantial activation of TAS2R10 by dapsone and chloroquine correlates with its high expression levels in porcine coronary arteries, underscoring its potential as a therapeutic target. However, not all TAS2R agonists demonstrated consistent effects with their corresponding receptor expression levels. The differential effects of TAS2R agonists, such as the potent vasorelaxant effect of flufenamic acid and the minimal response to denatonium benzoate in porcine coronary arteries, may be attributed to several factors. Species-specific differences in TAS2R subtype expression profiles, receptor-effector coupling, and downstream signaling pathways can significantly influence functional outcomes (Meyerhof et al., 2010). Additionally, tissue-specific expression and variations in endothelial versus smooth muscle signaling mechanisms could explain why denatonium has been reported to induce relaxation in the rat corpus cavernosum but exhibited limited effects in the porcine coronary model used in this study (Devillier et al., 2015).

Notably, flufenamic acid, which primarily targets TAS2R14 (Di Pizio et al., 2020; Hu et al., 2024), exhibited the most potent vasorelaxant effect, suggesting the involvement of TAS2R14. Phenanthroline, a selective TAS2R5 agonist (Grassin-Delyle et al., 2019) stimulates moderate vasorelaxation. However, TAS2R5 and TAS2R14 were not included in our qPCR panel because of the absence of available sequence information in genetic databases. Immunohistochemical studies were not performed because of the lack of specific antibodies against porcine TAS2R5 and TAS2R14. Moreover, selective antagonists for TAS2R5 and TAS2R14 are currently unavailable, limiting the ability to pharmacologically confirm TAS2R-mediated responses. This underscores the need for future research to characterize TAS2R5-and TAS2R14-mediated responses in coronary artery tissues.

Additionally, the high expression of TAS2R42, which currently lacks a selective agonist, opens a promising research avenue. The development of specific agonists for this subtype may reveal its role in coronary physiology (Bloxham et al., 2020; Tuzim and Korolczuk, 2021). The diverse effects observed with different agonists on overlapping TAS2R subtypes also indicate the potential of combination therapies that capitalize on synergistic effects. Future studies on the human TAS2R subtypes that mediate coronary vasorelaxation and the development of more potent and selective agonists are warranted. Such efforts could pave the way for innovative and targeted therapies for ischemic heart disease by leveraging the unique properties of TAS2Rs in the coronary arteries.

The use of porcine coronary artery models in this study has provided insights into TAS2R agonist-induced vasorelaxation but also has several limitations. First, the findings may not be directly translatable to humans owing to potential differences in TAS2R expression and cardiovascular physiology between porcine and human systems. Second, the limited set of TAS2R agonists explored suggests that more potent and selective compounds may have been overlooked. Third, the isolated coronary artery ring setup in an *ex vivo* environment may not capture the regulatory mechanisms present in intact organisms.

Although the flufenamic acid-induced relaxation aligns with TAS2R activation profiles, direct evidence for TAS2R14 involvement was not obtained. Future studies utilizing receptor-specific antagonists or genetic tools are warranted to clarify the causal role of specific TAS2R subtypes. In addition, residual endothelial cells may have contributed to the observed SK_Ca-mediated responses despite mechanical denudation. Some studies have reported low-level SK_Ca channel expression in vascular smooth muscle cells under certain physiological conditions (Jackson, 2017), which may have influenced the outcomes. Other pathways, such as cyclooxygenase (COX)-derived prostanoids, may also be involved and warrant further investigation.

Finally, while the present study highlights the therapeutic potential of TAS2R agonists for coronary vasorelaxation, further validation in human coronary arteries—particularly comparing healthy and diseased states—is critical. Future research should also comprehensively assess the therapeutic efficacy and safety of TAS2R-targeted strategies, and expand the repertoire of agonists to develop more selective and safer interventions.

## 5 Conclusion

This study demonstrated that TAS2R agonists, including flufenamic acid (a selective TAS2R14 agonist), phenanthroline (a selective TAS2R5 agonist) and chloroquine, induced concentration-dependent vasorelaxation in porcine coronary arteries. The mechanisms underlying flufenamic acid-induced vasorelaxation may involve potassium channels and NO signaling. The expression of the TAS2R subtypes 42, 40, 10, and 38 in these arteries suggests potential therapeutic targets for the management of coronary artery diseases. Thus, TAS2Rs may play an important role in the relaxation of porcine coronary arteries.

## Data Availability

The original contributions presented in the study are included in the article/Supplementary Material, further inquiries can be directed to the corresponding author.
