# Supplementary material for: Bitter taste receptor agonists induce vasorelaxation in porcine coronary arteries
Source: Front Pharmacol. 2025 Jul 7;16:1578913. doi: 10.3389/fphar.2025.1578913 (PMC12277086; doi:10.3389/fphar.2025.1578913)
Supplement: Supplementary file 1 [file Table1.docx]

**Supplemental Material**

The following primer sequences were used for amplifying and analyzing the expression of various TAS2R subtypes in porcine coronary artery samples:

pTAS2R3

Forward: 5'-TACTGCCTGAAAGTCGCCAG-3'

Reverse: 5'-TCAGTCACGTTGCCTGATCC-3'

pTAS2R4

Forward: 5'-CGTCACCACTCTCCTGTACG-3'

Reverse: 5'-TGATGACCAAGGAAGCAGCA-3'

pTAS2R7

Forward: 5'-ACCAGGCGGATGCAATTCAA-3'

Reverse: 5'-GGAGGTGGCTACAAGATAGGC-3'

pTAS2R8

Forward: 5'-GACTGCATCCTCGCCAGTTT-3'

Reverse: 5'-GAAGACACTGAGGCAGGTGG-3'

pTAS2R9

Forward: 5'-ACTTTATGCCACAGGGTCCA-3'

Reverse: 5'-GAGGGATCAGAAAGCTAGCGG-3'

pTAS2R10

Forward: 5'-GCACAACAGGCAGATGCAAT-3'

Reverse: 5'-GACCCCAAGGAAAAATGGCTG-3'

pTAS2R16

Forward: 5'-TGTGCTCGAGTGCTTGGTAA-3'

Reverse: 5'-AGGCTGGTGAGAATCACGTC-3'

pTAS2R38

Forward: 5'-TTTTGGGACGTGGTGAGGAG-3'

Reverse: 5'-ACCACAGGCCAGCTTGATTT-3'

pTAS2R40

Forward: 5'-TGAGGCTGTCGTTGTTCTCC-3'

Reverse: 5'-CCACGTTGGTCCCAGAGAAG-3'

pTAS2R41

Forward: 5'-AGAAGGCCAGCAGTGCTTAG-3'

Reverse: 5'-TTCCCAGTAGGCAGTCGAGA-3'

pTAS2R42

Forward: 5'-GTTGGTGGCTTTGTTGGACTC-3'

Reverse: 5'-GCTTAAGCAGGTGGAAAGCC-3'

pTAS2R60

Forward: 5'-TGTGTTTCCATCCCAGGAGC-3'

Reverse: 5'-TCAGCCTGCGGTTACTCAAG-3'

All primers were designed to specifically amplify the corresponding porcine TAS2R subtype sequences. Primer sequences are provided in the 5' to 3' direction.
